# Immunomodulatory and Antimicrobial Effects of Pd(II)-Pincer-Type Complex

**DOI:** 10.3390/biomedicines14061200

**Published:** 2026-05-27

**Authors:** Zorana Maric Ostovic, Katarina Mijacic, Isidora Kostic, Nevena Gajovic, Milena Jurisevic, Bojana Simovic Markovic, Vladimir Markovic, Sanja Zornic, Snezana Jovanovic Stevic, Bojan Kujundzic, Srdjan Masic, Dragana Drakul, Ivan Jovanovic

**Affiliations:** 1 Faculty of Medicine, University of East Sarajevo, 73300 Foca, Bosnia and Herzegovina; 2 Center for Molecular Medicine and Stem Cell Research, Faculty of Medical Sciences, University of Kragujevac, 34000 Kragujevac, Serbia; 3 Department of Pharmacy, Faculty of Medical Science, University of Kragujevac, Svetozara Markovića 69, 34000 Kragujevac, Serbia; 4 Institute of Public Health Kragujevac, 34000 Kragujevac, Serbia

**Keywords:** palladium(II) complex, immunomodulation, splenocytes, cytokines, antimicrobial activity

## Abstract

**Background:** The newly synthesized palladium(II) complex [Pd(L1)Cl]Cl (where L1 = *N*^2^,*N*^6^-bis(5-methylhthiazol-2-yl)pyridine-2,6-dicarboxamide) has demonstrated significant in vitro antitumor activity. In this study, the effects of this complex on the immune response and its antimicrobial potential were evaluated. **Methods**: Splenocytes isolated from mice were treated with lipopolysaccharide (LPS)/Concanavalin A (ConA) along with the Pd(II) complex. The concentrations of IFN-γ, IL-17, IL-4, TNF-α, IL-1β, and IL-10 were measured using commercial ELISA kits. The antimicrobial effect was tested against reference strains of Gram-positive and Gram-negative bacteria, as well as yeast. The Minimal Inhibitory Concentration (MIC) was determined via the broth microdilution method, followed by the determination of Minimal Bactericidal/Fungicidal concentrations (MBC/MFC). **Results:** The Pd(II) complex induced an increase in the concentration of all tested cytokines compared to untreated cells. Co-treatment with Pd(II) complex and LPS significantly increased the levels of IFN-γ, IL-1β, and IL-17 compared to the LPS-only-stimulated group. Co-treatment with ConA and the Pd(II) complex resulted in a significant increase in TNF-α and IL-17 levels, whereas a significant decrease was observed in the concentrations of IL-10, IL-4, and IFN-γ compared to the ConA-only-stimulated group. The tested complex showed weak to moderate antimicrobial activity, Gram-positive bacteria showed better susceptibility to the examined complex compared to Gram-negative. **Conclusions:** Results of the study indicate that the Pd(II) complex exhibits a significant immunomodulatory effect on splenocytes, alongside weak to moderate antimicrobial activity.

## 1. Introduction

Two leading problems of modern medicine are the increasing incidence of malignant diseases and the growing resistance of microorganisms to available antimicrobial drugs [1,2,3,4]. Recent WHO surveillance indicates that approximately one in six bacterial infections worldwide in 2023 were resistant to antibiotic treatment, and resistance trends are increasing in over 40% of monitored pathogen–drug combinations [5]. This indicates an urgent need to find new drugs, which will be more effective than those currently used in the treatment of tumors and infections. Potential candidates include transition metal compounds such as platinum and palladium [6,7,8,9,10,11]. Cisplatin and its analogues have been used as chemotherapeutics for years, but their use is limited due to the appearance of side effects and tumor resistance to them [12,13,14]. Palladium(II) complexes currently are not clinically approved, but they have attracted increasing attention as potential anticancer agents due to their structural and mechanistic similarity to cisplatin. However their significantly higher ligand exchange rates often result in reduced kinetic stability and uncontrolled reactivity under physiological conditions, which has so far limited their clinical application. Despite these challenges, recent studies demonstrated that appropriate ligand design—particularly the use of chelating and pincer-type frameworks—can effectively modulate palladium reactivity, enhancing stability while preserving biological activity [15]. Moreover, Pd(II) complexes have shown promising DNA-binding properties, protein interactions, and the abilities to induce apoptosis in cancer cells, often with improved selectivity compared to platinum analogues [16,17]. These findings highlight palladium complexes as a valuable platform for the development of novel anticancer agents with tunable pharmacological profiles. In addition to the antitumor effects of transition metal compounds, increasing attention is on their ability to modulate the immune response [17,18]. In the context of oncology, by modifying the immune response or modifying the tumor microenvironment, drugs can enhance or restore the ability of the immune system to recognize and eliminate the tumor [19]. In other words, by acting on the immune system, drugs should stop the mechanisms by which tumors avoid the immune response [20]. Although research is still at an early stage, the results obtained so far with palladium compounds indicate their potential for further research and clinical application.

The growing resistance of microorganisms to available antimicrobial drugs indicate the need to find new drugs [21,22]. Some infections that were previously easily treated now lead to serious conditions and even fatal outcomes. Palladium complexes and nanoparticles have shown significant antimicrobial activity against a wide range of microorganisms: bacteria, fungi and viruses [9,23,24,25]. Mechanisms of the antimicrobial effect of complexes with Pd include: damage to the cell membrane, causing oxidative stress and impact on the genetic material of microorganisms [26].

**Figure 1:**
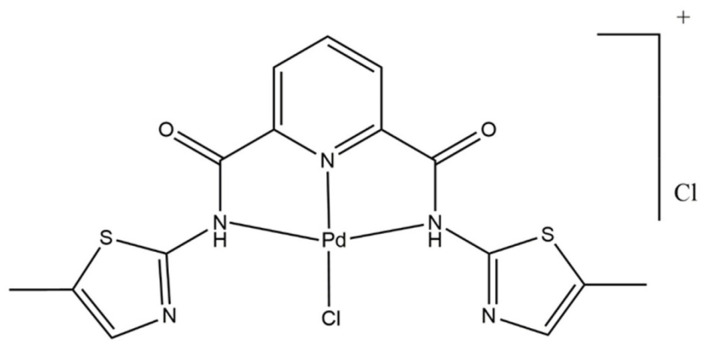
The structure of [Pd(*N*^2^,*N*^6^-bis(5-methylthiazol-2-yl)pyridine-2,6-dicarboxamide)Cl]Cl complex.

The previous study showed an antitumor effect of palladium(II) complex [Pd(*N*^2^,*N*^6^-bis(5-methylthiazol-2-yl)pyridine-2,6-dicarboxamide)Cl]Cl (Figure 1). The biological activity of this complex is governed by the rational design of its tridentate pincer-type ligand and the coordination environment of the Pd(II) center. The chelating ligand enhances complex stability and modulates the inherently high reactivity of Pd(II), enabling controlled nucleophilic substitution under physiological conditions. Structurally, the presence of methylthiazole moieties contributes to less disruptive interactions with DNA, compared to classical intercalators. Therefore, the present study aimed to further investigate their potential immunomodulatory and antimicrobial effects in vitro [27].

## 2. Materials and Methods

### 2.1. Isolation and Treatment of Splenocytes

The complex [Pd(*N*^2^,*N*^6^-bis(5-methylthiazol-2-yl)pyridine-2,6-dicarboxamide)Cl]Cl was synthesized according to the published procedure [27]. To the solution of K_2_PdCl_4_ (100 mg, 0.306 mmol) in 10 mL of dimethylformamide (DMF) was added 1 eq. of L1 ligand (110 mg) dissolved in 10 mL of DMF. The mixture was refluxed overnight and the obtained clear yellow solution was filtered and concentrated using a rotary evaporator. The volume was reduced under vacuum to 5 mL and upon adding of 20 mL of ether to the solution, pale yellow precipitate of complex was obtained. The precipitate was filtered, washed with water, ethanol and ether and left to air dry [27].

Yield (118 mg, 72%). Anal. Calc. for PdCl_2_N_5_S_2_O_2_C_15_H_13_ (%) (MW = 536.75): N, 13.05; C, 33.56; H, 2.44. Found: N, 12.98; C, 33.43; H, 2.43. IR (KBr, ν, cm^−1^): 3433.88 (N–H stretch); 3079.25, 3023.23 (C=C–H stretch); 2976.95–2779.15 (C–H stretch); 1635.18 (C=O stretch); 1461.99 (C=N stretch). ^1^H NMR characterization (DMSO-d_6_, 200 MHz). ^1^H NMR (δ, ppm): 11.50 (s, 2H, NH); 7.50–7.35 (m, 3H, py–CH_meta/para_), 6.37 (s, 2H, thiazole–CH), 1.48 (s, 6H, CH_3_). LDI TOF MS: *m*/*z* = 501.1 corresponds to complex cation formed by loss of labile Cl^−^ ligand [Pd − Cl^−^]^+^ [27].

The spleen, isolated from euthanized mice, was carefully dissected under sterile conditions. The organ was rinsed in Phosphate-Buffered Saline (PBS) and then transferred to a Petri dish with 5 mL of RPMI-1640 medium. The spleen was mechanically dispersed and forced gently through a 40 µm cell strainer nylon mesh using a sterile syringe plunger to remove aggregates and tissue debris. The cell suspension was centrifuged at 1500 rpm for 5 min at 4 °C. After removing the supernatant, the cell pellet was resuspended in 2 mL erythrocytes lysis buffer (RBC lysis buffer) on ice for 5 min to remove red blood cells. Lysis was stopped by adding 8 mL of RPMI-1640 medium. The cell suspension was centrifuged again (1500 rpm, 5 min, 4 °C), and the pellet was resuspended in final volume of complete medium (RPMI- 1640 with 10% fetal bovine serum (FBS), 2 mmol/L L-glutamine, 1 mmol/L penicillin, and 1 mmol/L streptomycin). The total cell count and viability were determined using a hemocytometer and trypan blue assay [18]. The cells were placed in 96-well microtiter plates (2 × 10^5^ cells/well), and divided into 6 different groups: a group of cells incubated with medium only (control), a group treated with Concanavalin A—ConA (0.5 µg/mL), a group treated with *Escherichia coli* lipopolysaccharide—LPS (0.5 µg/mL), a group treated with Pd(II) complex, a group simultaneously treated with ConA and Pd(II) complex, and a group simultaneously treated with LPS and Pd(II). The concentration of Pd(II) complex used in all experiments was previously determined according to the IC_50_ value. The microtiter plate was then incubated for 24 h at 37 °C in humidified atmosphere of 5% CO_2_ and 95% air.

### 2.2. Cytokine Analysis

After the incubation period, supernatant from all samples were collected after centrifugation (1500 rpm, 10 min, 4 °C) to remove cell debris. The concentration of cytokines IFN-γ, IL-17, IL-4, TNF-α, IL-1β, and IL-10 were determined from the supernatant using commercially available ELISA test from R&D Systems (Minneapolis, MN, USA), according to the manufacturer’s instructions. Absorbance was measured at 450 nm, and cytokine concentrations were calculated based on the standard curve.

### 2.3. Reference Strains of Microorganisms

The following reference strains of microorganisms were used to test the antimicrobial activity of the complex: *Staphylococcus aureus* (ATCC 25923), *Staphylococcus epidermidis* (ATCC 12228), *Staphylococcus saprophyticus* (ATCC 15305), *Listeria monocytogenes* (ATCC 19115), *Streptococcus pneumoniae* (ATCC 49619), *Enterococcus faecalis* (ATCC 29212), *Escherichia coli* (ATCC 25922), *Proteus mirabilis* (ATCC 25933), *Candida albicans* (ATCC 10231) and *Saccharomyces cerevisiae* (ATCC 9763).

### 2.4. Examination of the Antimicrobial Effect of Pd(II) Complex

The Minimal Inhibitory Concentration (MIC) of the complex was determined by the broth micodilution method, followed by the Minimal Bactericidal/Fungicidal Concentration (MBC/MFC). MIC, MBC and MFC values for commonly used antimicrobial drugs were determined for all tested microorganisms, in order to compare them with the standards, prescribed by the European Committee on Antimicrobial Susceptibility Testing (EUCAST), and verify that the susceptibility of the tested strains is within the limits of the reference values. The microorganism suspension was prepared by taking 2–3 bacterial colonies, or yeast colonies, with a sterile loop from a solid medium after 24 h of incubation. The microorganisms were resuspended in saline solution to a density of 0.5 McFarland, which corresponds to an initial bacterial concentration of 10^8^ CFU/mL and a fungal concentration of 10^6^ CFU/mL. Dilutions were then made to a final concentration of 5 × 10^5^ CFU/mL. An amount of 0.1 mL of Mueller-Hinton broth (Liophilchem, Italy) was added to each well of the microtiter plate. Additionally, 0.1 mL of the tested complex was added to the first row of wells (initial concentration 2000 µg/mL), and two-fold serial dilutions were prepared. Two columns serve as controls: one for control antibiotic or antifungal agent and the other for microorganism growth control. Vancomycin was used as the control antibiotic for Gram-positive bacteria, Ceftriaxone for Gram-negative bacteria, and Fluconazole for fungi. Then, 10 µL of the bacterial/fungal suspension was added to each well of microtiter plate. After 24 h of incubation at 37 °C under aerobic conditions for bacteria, and 48 h for fungi, the results were read using an inverted mirror placed beneath the microtiter plate. This method enhanced contrast and facilitates the detection of bacterial growth. Additionally, for detection of growth of the microorganisms resazurin solution was used (Sigma Aldrich, Munich, Germany). In each well of the microtiter plate, 10 µL of 1 M resazurin solution was added to and incubation was extended for an additional 3 h. Resazurin is a blue compound that, in presence of oxidoreductase from living cells, is reduced to resorufin, which is pink [28]. The concentration of the complex in the first well where the content is clear or the well that remained blue was defined as the MIC. Then 10 µL of the solution from the remaining wells with higher complex concentrations were inoculated on Blood Agar with 5% sheep blood (Oxoid, Germany), or Sabouraud-dextrose agar for fungi. The plates were incubated for 24 h for bacteria, and 48 h for fungi. The MBC/MFC values is the concentration in the suspension used to inoculate the first plate with no growth of bacterial/fungal colonies, i.e., the first sterile plate.

### 2.5. Statistical Analysis

The SPSS software package version 23.0 (Statistical Package for Social Sciences SPSS 23.0 Inc., Chicago, IL, USA) was used for statistical data processing. For samples with a normal distribution, statistical significance was determined using Student’s *t*-test and for samples without a normal distribution, the Mann–Whitney U. The data are presented as mean ± standard error of the mean (SEM). The difference was considered significant when *p* < 0.05.

## 3. Results

### 3.1. Pd(II) Complex Increases Pro-Inflammatory Cytokine Concentrations

**Figure 2:**
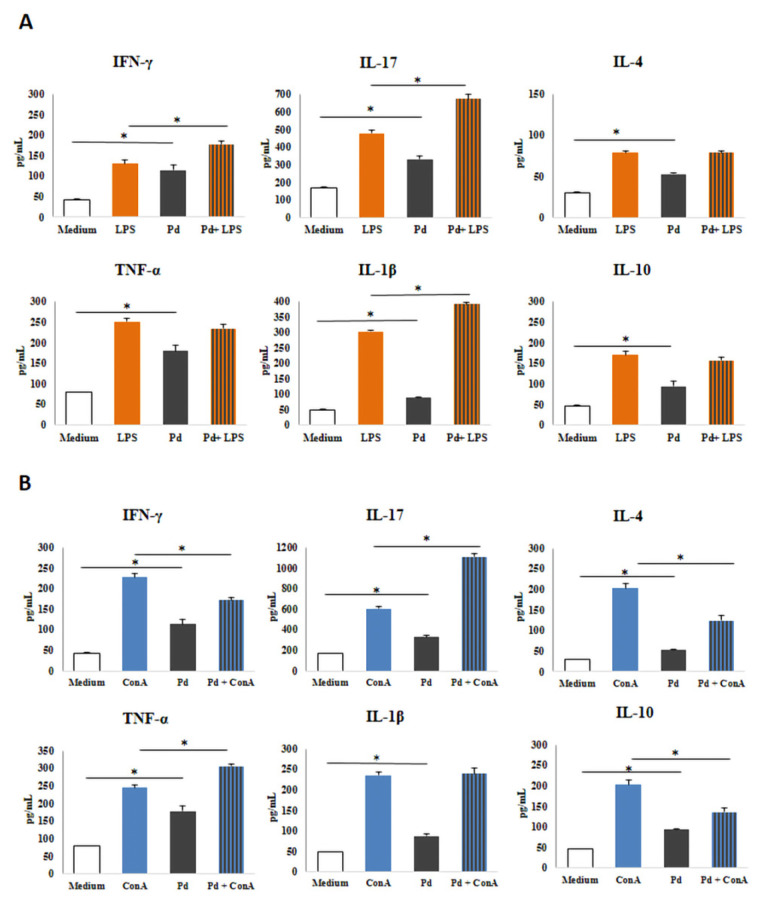
Cytokine concentration in supernatants of splenocytes stimulated with LPS or ConA and treated with Pd(II) complex. Section (**A**) displays the concentration of cytokines produced by splenocytes incubated with medium only, LPS, Pd(II) complex or a combination of Pd(II) complex and LPS. Section (**B**) displays the concentration of cytokines produced by splenocytes incubated with medium only, ConA, Pd(II) complex or a combination of Pd(II) complex and ConA. The concentration of IFN-γ, IL-17, IL-4, TNF-α, IL-1β and IL-10 were determined by ELISA method after 24 h incubation. Data are presented as mean ± SEM, and statistical significance was determined using Student’s *t*-test or Mann–Whitney U test, where appropriate (* *p* < 0.05).

LPS/ConA-priming increased the production of IFN-γ, IL-17, IL-4, TNF-α, IL-1β, and IL-10 compared to untreated cells (*p* < 0.05; Figure 2). Incubation with Pd(II) complex alone, increased the concentration of all tested cytokines compared to splenocytes incubated in the medium only (*p* < 0.05; Figure 2). Co-treatment with Pd(II) complex and LPS significantly increased concentration of IFN-γ, IL-17, and IL-1β (*p* < 0.05) compared to splenocytes treated with LPS only (Figure 2A). Also, co-treatment reduced TNF-α and IL-10 but without statistical significance, while the value of IL-4 remained unchanged in comparison to the LPS-stimulated group (Figure 2A).

Furthermore, co-treatment with the ConA and Pd(II) complex significantly increased TNF-α and IL-17 (*p* < 0.05) concentrations, while the increase in IL-1β did not reach statistical significance compared to the ConA-simulated group (Figure 2B). The same co-treatment induced a statistically significant decrease in the concentration of IL-10, IL-4, as well as IFN-γ (*p* < 0.05) compared to the ConA-primed group (Figure 2B).

### 3.2. Pd(II) Complex Elevates Ratio of Pro-Inflammatory Cytokines in Non-Activated to Activated Cells

**Figure 3:**
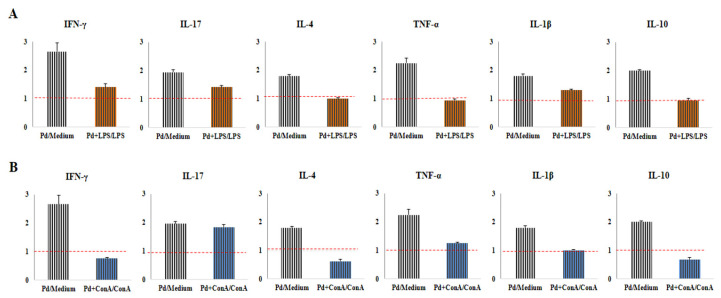
Ratio of cytokine concentrations in non-activated and LPS/ConA-activated cells treated with Pd(II) complex. Section (**A**) displays the ratio of cytokine concentrations in splenocytes treated with Pd(II) complex relative to untreated cells, and in cells co-treated with Pd(II)+LPS relative to LPS only. Section (**B**) displays the ratio of cytokine concentrations in splenocytes treated with Pd(II) complex relative to untreated cells, and in cells co-treated with Pd(II)+ConA relative to ConA only. The concentration of tested cytokines were determined by ELISA method after 24 h incubation. Data are presented as mean ± SEM, and statistical significance was determined using Student’s *t*-test or Mann–Whitney U test, where appropriate (*p* < 0.05). The red dashed line represents the baseline ratio of 1.

Pd(II) complex treatment significantly increased ratios of TNF-α, IL-4, and IL-10 in cells incubated in medium only (Pd/medium) in comparison to LPS-primed cells (Pd+LPS/LPS) (*p* < 0.05; Figure 3A). The elevation was also detected in IFN-γ, IL-17 and IL-1β levels but the difference did not obtain statistical significance (Figure 3A). Incubation with Pd(II) complex elevated the ratios of IFN-γ, IL-4, IL-1β, and IL-10 in untreated cells (Pd/medium), compared to the ConA-activated group (Pd+ConA/ConA) (*p* < 0.05; Figure 3B). The increase was detected in IL-17 and TNF-α levels but the difference did not reach statistical significance (Figure 3B).

### 3.3. Pd(II) Complex Increases Ratio of Pro-Inflammatory to Anti-Inflammatory Cytokine Concentration

**Figure 4:**
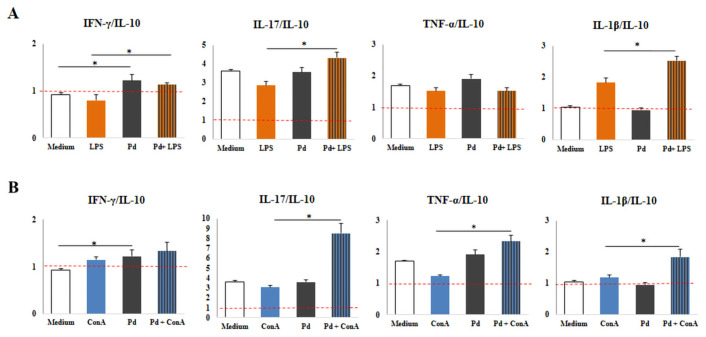
Pro- and anti-inflammatory cytokine ratios in supernatants of splenocytes. Section (**A**) shows cytokine levels in cells incubated with medium only, LPS, Pd(II) complex or a combination of Pd(II) complex and LPS. Section (**B**) shows cytokine levels in cells incubated with medium only, ConA, Pd(II) complex or a combination of Pd(II) complex and ConA. The concentration of tested cytokines was determined by ELISA method after 24 h incubation. Data are presented as mean ± SEM, and statistical significance was determined using Student’s *t*-test or Mann–Whitney U test, where appropriate (* *p* < 0.05).

Treatment with the Pd(II) complex significantly increased the ratio of IFN-γ/IL-10 in comparison to the untreated control (*p* < 0.05; Figure 4A). Also, co-cultivation with the Pd(II) complex and LPS significantly enhanced the ratio of IL-17/IL-10, TNF-α/IL-10, and IL-1β/IL-10 compared to LPS-stimulated splenocytes (*p* < 0.05; Figure 4A). Furthermore, co-treatment with the Pd(II) complex and ConA significantly elevated the ratio of IL-17/IL-10, TNF-α/IL-10, and IL-1β/IL-10 in comparison to ConA-primed cells (*p* < 0.05; Figure 4B).

### 3.4. Antimicrobial Effect of Pd(II) Complex

The Pd(II) complex showed weak to moderate antimicrobial activity, which varied depending on the tested bacterial strain. Gram-positive bacteria showed better susceptibility to the Pd(II) complex compared to Gram-negative (Table 1). The tested strains of Gram-negative bacteria: *Escherichia coli* and *Proteus mirabilis* were weakly sensitive to the Pd(II) complex with MIC and MBC values greater than 1000 μg/mL. *Staphylococcus epidermidis* had the highest susceptibility to the Pd(II) complex with MIC and MBC 15.63 μg/mL. The Pd(II) complex showed moderate susceptibility against *Staphylococcus saprophyticus* and *Streptococcus pneumoniae* with MIC and MBC values of 31.25 μg/mL, as well as against *Listeria monocytogenes* whose MIC and MBC were 62.5 μg/mL. Susceptibility of *Staphylococcus aureus* and *Enterococcus faecalis* was weak with MIC and MBC values greater than 1000 μg/mL. The Pd(II) complex displayed a weak antifungal effect on the yeast *Candida albicans* with MIC values of 500 μg/mL, and MFC 1000 μg/mL. The Pd(II) complex also demonstrated a weak antifungal effect on *Saccharomyces cerevisiae*, but better than *Candida albicans,* with MIC and MFC values of 125 μg/mL.

## 4. Discussion

Palladium-based drugs represent a promising base for anticancer agents. Palladium complexes have similar chemical and physical properties as their platinum analogs, but are significantly more reactive, up to 10^5^ times, which requires stabilization with appropriate ligands [29]. Compared to platinum-based drugs, palladium complexes, are increasingly investigated as potential alternatives due to their tunable reactivity and possibility of achieving improved selectivity through rational ligand design. While some Pd(II) complexes have demonstrated reduced toxicity toward normal cells in vitro, their overall toxicological profile is highly dependent on ligand architecture and kinetic stability, and thus cannot be generalized [6,29]. Comparative studies have shown that Pd(II) complexes can exhibit cytotoxic activity comparable to that of structurally analogous Pt(II) compounds, although the extent of this activity is highly dependent on ligand design and coordination environment. It was demonstrated that certain Pd(II) complexes achieve similar or even lower IC_50_ values than their platinum counterparts in selective cancer cell lines [30]. This finding supports the notion that Pd-based compounds can retain significant anti-proliferative potential despite their higher kinetic lability. The variability in activity underscores the critical role of ligand architecture in modulating both cytotoxicity and selectivity, indicating that rational ligand design is essential for optimizing the therapeutic profile of Pd(II) complexes.

The toxicity profile and distribution to target tissues of Pd(II) complexes can also be modulated by physical approaches such as advanced drug-delivery systems, including liposomal, nanoparticle-based, or albumin-bound formulations [28]. Nanoencapsulated Pd did not exhibit systemic toxicity in mice and was associated with preserved organ function, stable hematological parameters, and reduced distribution to critical organs such as the heart and bone marrow [31]. Although the investigated Pd(II) complexes have so far been evaluated only in solution, the final pharmaceutical formulation has not yet been established, since the present study represents an early stage of preclinical development. Considering the similarities with currently used metal-based anticancer drugs, future therapeutic applications would most likely initially involve parenteral administration, particularly intravenous formulations. Further studies evaluating the in vivo efficacy and toxicity, and pharmacokinetic properties of Pd(II)-pincer-type complex are necessary. It should also be considered that certain palladium-containing compounds have been associated with hypersensitivity reactions, including skin and respiratory irritation, particularly after occupational or prolonged exposure. However, such effects strongly depend on the chemical form, ligand environment, exposure route, and bioavailability of the palladium drugs, emphasizing the importance of future in vivo toxicological evaluation [32]. In addition to potential antitumor applications, topical formulations could also be considered for the antimicrobial use of these complexes, depending on future efficacy and safety evaluations [26].

Experimental studies demonstrate that Pd(II) complexes exert cytotoxic effects through DNA bindings, as well as the induction of oxidative stress and apoptotic pathways [33]. Recent in vivo studies conducted on murine models have demonstrated that dinuclear Pd(II) complexes, such as those involving spermine ligands, exhibit a biphasic pharmacokinetic profile with significant accumulation in the kidneys and liver, potentially leading to dose-dependent hepatorenal toxicity. Furthermore, contemporary research highlights that palladium(II) chloride and its organic derivatives can trigger endoplasmic reticulum stress and inflammation, mediated by the downregulation of antioxidant enzymes and the elevation of lipid peroxidation markers [34,35]. However, despite the growing number of studies, systematic dates regarding the in vivo toxicity and long-term safety of these compounds remain limited. Besides their direct effect on in vitro and in vivo cancer models, it is proven that palladium agents have potent immunomodulatory properties [17,36]. Our recent study has revealed that a novel Pd(II) complex, [Pd(*N*^2^,*N*^6^-bis(5-methylthiazol-2-yl)pyridine-2,6-dicarboxamide)Cl]Cl, exhibited significant dose-dependent cytotoxicity across human and mouse cell lines of colorectal and breast cancer, while simultaneously demonstrating favorable selectivity by maintaining relatively low toxicity toward non-cancerous mesenchymal stem cells (mMSCs). Notably, the Pd(II) complex displayed potent anti-proliferative activity comparable to the clinical standard, cisplatin, against both HCT116 and CT26 colon cancer cells, even surpassing cisplatin’s efficacy at lower concentration ranges (3.9–15.625 µM). The observed variations in sensitivity, such as the heightened response of human MDA-MB468 cells to Pd(II) compared to 4T1 cells, suggest that the cytotoxic potential of these complexes may be influenced by specific tumor-type characteristics and species-specific cellular environments. The superior selective cytotoxicity of the Pd(II) is mediated by its ability to orchestrate tumor-specific apoptosis through the modulation of the Bax/Bcl-2 ratio and caspase-3 activation, while simultaneously inducing S-phase cell cycle arrest and suppressing pro-survival *p*-AKT signaling [27]. Because of these results, our further research was focused on potential immunomodulatory and antimicrobial effects of this complex in vitro.

Our first goal was to assess cytokine production in splenocytes obtained from healthy mice, after LPS/ConA stimulation, incubation with Pd(II) complex, and LPS/ConA+Pd(II) complex co-treatment. LPS is a component of the outer membrane of Gram-negative bacteria. It is released during their breakdown, hence its name endotoxin. Lipid A is the carrier of toxic activity and can cause a strong inflammatory reaction in the organism [37,38]. Its application is particularly important in the context of innate immune response research, where cells are non-specifically activated through recognition by Toll-like receptor-4 (TLR-4) [39]. ConA is a protein from the lectin group, which mainly binds to carbohydrates, primarily mannose and glucose. It is a potent mitogen that activates T lymphocytes cells in the adaptive immune response, and stimulates the release of cytokines [40]. In our research, LPS was applied to induce activation of innate immune cells, and ConA was used to activate the cells of adaptive immunity. As expected, the stimulation with LPS/ConA significantly increased concentration of all tested cytokines, compared to cells incubated in the medium only (Figure 2). Also, treatment with the Pd(II) complex alone increased the concentrations of all examined cytokines compared to untreated splenocytes (Figure 2). Co-treatment with the Pd(II) complex and LPS significantly elevated the concentrations of IFN-γ, IL-1β, and IL-17 compared to LPS-stimulated splenocytes. Moreover, Pd(II)+ConA co-treatment significantly elevated concentrations of pro-inflammatory TNF-α, IL-17 and IL-1β (did not obtain statistical significance) while simultaneously reducing concentrations of anti-inflammatory IL-4 and IL-10 compared to ConA-activated cells (Figure 2). Next we analyzed the ratio of non-activated to activated innate/adaptive immune cells and demonstrated that the production of all tested cytokines was higher in non-activated splenocytes in comparison to activated cells (Figure 2). We then evaluated the ratio between pro-inflammatory and anti-inflammatory cytokines produced by innate and adaptive immune cells. These ratios were calculated by dividing the concentration of each measured pro-inflammatory cytokine by the concentration of the anti-inflammatory cytokine IL-10. Results revealed that the tested Pd(II) complex predominantly promotes pro-inflammatory cytokines IFN-γ and TNF-α over anti-inflammatory IL-10 in both non-activated and activated cells (innate and adaptive immunity cells) (Figure 4). These results suggest that the Pd(II) complex exhibits its immunomodulatory effects by shifting the cytokine production toward a pro-inflammatory profile. While the effect is more pronounced in non-activated innate cells, analysis of cytokine ratios also shows that activated cells, both innate and adaptive, exhibit a similar trend, indicating that the Pd(II) complex can enhance pro-inflammatory responses across different immune activation stages. In line with our results, Jakovljevic et al. demonstrated that the Pd(II) complex with ethyl derivate of thiosalicylic acid ligand exhibits immunomodulatory effects especially on non-activated splenocytes of both innate and adaptive immunity by enhancing pro-inflammatory, and reducing anti-inflammatory cytokine levels [17]. Also, Reale et al. showed that palladium salt inhibited the release of IFN-γ and IL-10, whereas palladium nanoparticles enhanced IFN-γ release and inhibited TNF-α secretion in peripheral blood mononuclear cells stimulated with LPS [41]. Overall, our results indicate that the Pd(II) complex has the potential to modulate the immune response by promoting pro-inflammatory cytokine production, mainly in non-activated cells. As most human tumors are poorly immunogenic, they do not induce a potent acquired immune response, and components of innate immunity play a major role in antitumor immunity. Taking this into account, the resulting phenomenon highlights the potential of the Pd(II) complex not only as an agent that directly acts on tumor cells but also as an enhancer of anti-tumor immunity.

Due to the rising resistance of microorganisms to existing treatments, heavy metal complexes like palladium have gained attention as promising antimicrobial agents over recent years [42]. Furthermore, the development of novel Pd(II) complexes remains of considerable interest due to the possibility that structural modifications could yield compounds with biological and toxicity profiles distinct from those associated with conventional antimicrobial or platinum-based agents. Numerous clinically used antimicrobial agents, including aminoglycosides, vancomycin, and certain antifungal drugs, are also associated with certain renal adverse effects including acute kidney injury, tubular damage, and interstitial nephritis [43]. Similarly, clinically used platinum compounds, particularly cisplatin, are well known for their dose-limiting nephrotoxicity, which represents one of a major challenge of metal-based chemotherapy. Although Pd(II) complexes are often considered potentially less nephrotoxic than conventional platinum compounds due to differences in coordination chemistry this assumption requires careful experimental confirmation through dedicated in vivo safety studies [44]. Our results demonstrated that the Pd(II) complex has weak to moderate antimicrobial activity. The sensitivity to the Pd(II) complex was higher in Gram-positive compared to Gram-negative bacteria, with the exception of *Staphylococcus aureus* and *Enterococcus faecalis*, which exhibited high MIC values. Furthermore, a stronger antimicrobial effect was observed against fungi compared to Gram-negative bacteria. *Saccharomyces cerevisiae* exhibited greater susceptibility to the Pd(II) complex compared to *Candida albicans*. Similarly, Ćorović et al. proved that a newly synthesized Pd(II) complex with dialkyl esters of (S,S)-propylenediamine-N,N’-di- (2,2′-di-(4-hydroxy-benzil))acetic acid shows selective and moderate antimicrobial activity [45]. In addition, various Pd(II) complexes have demonstrated strong antimicrobial activity against Gram-positive and Gram-negative bacteria, as well as yeasts [46,47,48]. The antimicrobial activity of the Pd(II) complex can be attributed to mechanisms commonly observed for metal-based compounds. These include interactions with microbial DNA and proteins, potentially interfering with replication and essential enzymatic processes [22,49,50]. In addition, it is proven that palladium complexes can induce the generation of reactive oxygen species, leading to oxidative stress and cell damage [50]. The greater sensitivity of Gram-positive bacteria may be related to their simpler cell wall structure compared to Gram-negative bacteria, which possess an additional outer membrane barrier [49]. Although these mechanisms were not directly examined in this study, they are consistent with literature reports and provide a plausible explanation for the observed activity [22,26,42,49,50]. Taken together, these results indicate that the Pd(II) complex exhibits weak to moderate antimicrobial activity against various microorganism species.

## 5. Conclusions

Our results demonstrated a significant immunomodulatory effect of newly synthesized mononuclear Pd(II) complex, [Pd(*N*^2^,*N*^6^-bis(5-methylthiazol-2-yl)pyridine-2,6-dicarboxamide)Cl]Cl, which acts more intensely on non-activated immune cells. This effect was primarily manifested in the stimulation of pro-inflammatory cytokine production. This may be particularly significant in the context of oncology and the reduction in immunosuppressive tumor microenvironments, especially since the newly synthesized Pd(II) complex also exhibits a substantial direct antitumor effect. Additionally, the results show that the Pd(II) complex possesses weak to moderate antimicrobial activity. Due to these effects, the Pd(II) complex may be a strong candidate for further investigations.

## Figures and Tables

**Table 1:** MIC, MBC and MFC values for antimicrobial agent and MIC, MBC and MFC values Pd(II) complex.

Tested Strains of Microorganisms	MIC, MBC and MFC Values (μg/mL) *					
	Vancomycin		Ceftriaxone		Pd(II) Complex	
	MIC	MBC	MIC	MBC	MIC	MBC
*Escherichia coli*	-	-	0.24	0.24	>1000	>1000
*Protesu mirabilis*	-	-	0.06	0.06	>1000	>1000

*Staphylococcus aureus*	0.98	1.95	1.95	3.91	>1000	>1000
*Staphylococcus epidermidis*	1.95	1.95	3.91	7.81	15.63	15.63
*Staphylococcus saprophyticus*	0.98	0.98	7.81	15.63	31.25	31.25
*Streptococcus pneumonia*	0.24	0.49	0.06	0.12	31.25	31.25
*Enterococcus faecalis*	1.95	1.95	-	-	>1000	>1000
*Listeria monocytogenes*	0.12	0.49	-	-	62.5	62.5

	Fluconazole				Pd(II) complex	
	MIC	MFC			MIC	MFC
*Candida albicans*	0.49	0.49	-	-	500	1000
*Saccharomyces cerevisiae*	0.49	0.98	-	-	125	125

* SD = 0.00 for all measurements.

## Data Availability

The data presented in this study are available on request from the corresponding author. The data are not publicly available due to established practices of the authors.

## Abbreviations

The following abbreviations are used in this manuscript:

LPS	lipopolysaccharide
ConA	Concanavalin A
Pd	palladium
MIC	Minimal Inhibitory Concentration
MBC	Minimal Bactericidal Concentration
MFC	Minimal Fungicidal Concentration
PBS	Phosphate-Buffered Saline
FBS	fetal bovine serum
ELISA	Enzyme-Linked Immunosorbent Assay
IL	Interleukin
IFN-γ	Interferon gamma
TNF-α	Tumor necrosis factor-alpha
EUCAST	European Committee on Antimicrobial Susceptibility Testing
SPSS	Statistical Package for Social Sciences
SEM	standard error of the mean
mMSCs	Multipotent mesenchymal stromal cells
TLR-4	Toll-like receptor-4
HCT116	Human Colorectal Carcinoma cell line
CT26	Murine Colon Carcinoma
MDA-MB468	Human Breast Adenocarcinoma
4T1	Murine Mammary Carcinoma
